# Raman Spectroscopy of Practical LIB Cathodes: A Study of Humidity-Induced Degradation

**DOI:** 10.3390/molecules30163448

**Published:** 2025-08-21

**Authors:** Claudio Mele, Filippo Ravasio, Andrea Casalegno, Elisa Emanuele, Claudio Rabissi, Benedetto Bozzini

**Affiliations:** 1 Dipartimento di Ingegneria dell’Innovazione, Università del Salento, via Monteroni, 73100 Lecce, Italy; claudio.mele@unisalento.it; 2 Dipartimento di Energia, Politecnico di Milano, via Lambruschini 4, 20156 Milano, Italy; filipporavasio.chimica@gmail.com (F.R.); andrea.casalegno@polimi.it (A.C.); elisa.emanuele@polimi.it (E.E.); claudio.rabissi@polimi.it (C.R.)

**Keywords:** Raman, battery, LIB, cathode, degradation, humidity

## Abstract

Exposure of LIB materials to ambient conditions with some level of humidity, either accidentally owing to imperfect fabrication or cell damage, or deliberately due to battery opening operations for analytical or recycling purposes, is a rather common event. As far as humidity-induced damage is concerned, on the one hand the general chemistry is well known, but on the other hand, concrete structural details of these processes have received limited explicit attention. The present study contributes to this field with an investigation centered on the use of Raman spectroscopy for the assessment of structural modifications using common lithium iron phosphate (LFP) and nickel–cobalt–manganese/lithium–manganese oxide (NCM-LMO) cathodes. The impact of humidity has been followed through the observation of differences in Raman bands of pristine and humidity-exposed cathode materials. Vibrational spectroscopy has been complemented with morphological (SEM), chemical (EDS), and electrochemical analyses. We have thus pinpointed the characteristic morphological and compositional changes corresponding to corrosion and active material dissolution. Electrochemical tests with cathodes reassembled in coin cells allowed for the association of specific capacity losses with humidity damaging.

## 1. Introduction

Lithium-ion batteries (LIBs) play a key role in contemporary modern energy storage systems, powering everything from portable electronics to electric vehicles and grid-scale applications. Central to their performance and longevity are the structural and electrochemical stabilities of their electrode materials, particularly the anode and cathode active phases. However, these materials are known to be sensitive to environmental conditions during handling, processing, and storage. Among the various external factors, ambient humidity has emerged as a critical contributor to irreversible degradation phenomena, including surface reactions, phase transformations, and the formation of passivating or parasitic compounds. These changes can detrimentally affect lithium transport, electronic conductivity, and interfacial stability, ultimately compromising cell efficiency and cycle life [1,2,3,4,5,6]. The interaction between water vapor and electrode materials is especially problematic for high-energy-density electrodes such as layered oxides, spinels, and silicon-based anodes. For instance, layered transition metal oxides (e.g., NMC, NCA) may undergo surface hydrolysis or carbonate formation, altering the surface chemistry and affecting subsequent solid electrolyte interphase (SEI) formation [7,8]. Similarly, moisture-induced oxidation or hydroxylation in anode materials can lead to gas evolution, volume changes, and electrode–electrolyte incompatibilities [9,10,11,12,13]. Despite increasing awareness of these issues, the precise mechanisms of humidity-induced degradation remain insufficiently understood, partly due to the complexity of coupled chemical, structural, and electrochemical processes. In particular, the literature includes pioneering studies on the effects of uncontrolled conditions during the opening process [14], with humidity and water effects on different cathode chemistries being extensively documented. Typically, after discharging the battery stack and separating individual cells, electrodes are exposed to air during the opening of pouch cells. LIB components, particularly the electrolytes, are highly sensitive to humidity. Water reacts with lithium salts such as LiPF_6_, producing acidic species like hydrofluoric acid (HF), which degrade the active materials [15]. Nickel–cobalt–manganese oxide (NCM) cathodes, for example, undergo complete de-lithiation during this process, accompanied by aluminum dissolution and pore occlusion in the electrodes, with the formation of aluminum-hydroxide-based compounds [2]. In the case of lithium iron phosphate (LFP) cathodes, the literature reports that long-term air exposure leads to significant oxidation of LiFePO_4_. Ferric ions form a disordered, partially hydrated phosphate phase, with an intermediate structure between olivine and Li_x_FePO_4_(OH)_x_ [16,17]. Another aspect of humidity-induced damage is exposure during disassembly of LIBs for post mortem assessments, that can cause additional damaging artefacts [18]. Material-handling protocols for cell dismantling are predominantly implemented manually at the laboratory scale. To address the limitations of manual disassembly and mitigate associated risks, researchers have developed automated systems for opening cells and separating materials. However, these systems often require customization to accommodate different battery types and sizes, resulting in high costs and limited throughput [19].

In the present paper, we concentrate on the role of Raman spectroscopy to pinpoint structural aspects of the exposure to humidity. In addition to the rich literature on the use of Raman spectroscopy for the study of LIBs [20,21,22], a close-knit group of papers has been published on the use of Raman spectroscopy to assess humidity-induced degradation of LIB electrode materials. As far as cathodes are concerned, the authors in [23] investigated water adsorption/desorption from LFP and [24] explored humidity exposure effects on Ni-rich layered oxide cathode materials, pinpointing basic salt formation and surface amorphization. Regarding solid electrolytes, the authors in [25] studied the degradation of Li_4_SnS_4_ in the presence of trace amounts of water. The available literature evidence shows changes in vibrational bands, consistent with surface amorphization, carbonate and hydrate phase formation, and altered interlayer bonding. Moreover, changes in the carbon band pattern resulting from humidity exposure have been presented in [26]. In the present study, to highlight the potential value of this approach, we have focused on cathode materials harvested from common commercial LIBs: in particular, lithium iron phosphate (LFP) and nickel–cobalt–manganese/lithium–manganese oxide (NCM-LMO). The specific aim of this work was to pinpoint the technical aspects related to ex situ analyses of practical electrodes, highlighting instrumental aspects, extraneous phase formation, active material modifications, and carbon modifications. To complement vibrational spectroscopy work, we have carried out morphological (SEM), chemical (EDS), and electrochemical characterizations.

## 2. Materials and Methods

### 2.1. Raman Spectroscopy

This work is centered on the use of Raman spectroscopy for the characterization of the vibrational structure of LIB cathode materials, in pristine conditions and after exposure to humidity. The chemical processes involved in the fabrication, operation, and degradation of LIB cathodes involve complex phenomena, such as bond breaking/formation, ion transport, structural changes, ionic coordination and transport, and the evolution of nano- and mesostructures that can be followed accurately and non-destructively by vibrational spectroscopy. Moreover, Raman spectroscopy, that employs visible wavelengths, lends itself ideally to in situ and in operando work. Moreover, the single most relevant contribution of vibrational spectroscopy is in following physico-chemical processes that cannot be probed by other characterization techniques, typically including structural processes consisting of changes in the chemical nature of moieties virtually without compositional changes, solvation, and coordination structures of all classes of electrolytes and interface-controlled electrochemical processes. In the present study, we have employed ex situ Confocal Spontaneous Raman Spectroscopy to probe the bulk molecular properties of practical LIB cathodes, incorporating carbon and a binder, in addition to the intercalation-active compound. The confocal optical configuration allows us—for the case relevant to this investigation— to confine spatially the probed volume, enabling the analysis of selected micro-regions; thanks to the methodology defined in this study, future studies will address the following: (i) sample mapping with lateral resolution in the micrometer range, (ii) the use of background-free stimulated Raman spectroscopy [27], and (iii) resonant Raman spectroscopy, by tuning the excitation wavelength [28].

**Figure 1:**
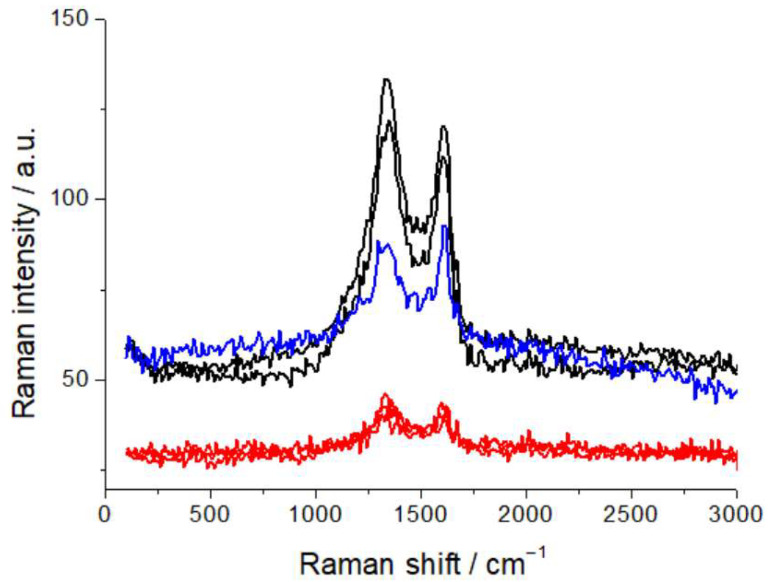
Raman spectra of graphite anodes, measured with D06 (black and blue plots) and D2 (red plots) optical densities with 6 min exposure to the beam, showing residual laser-induced fluorescence and beam damage with D06. For details, refer to the text.

For this study, the samples were laid horizontally on the translation stage of the confocal microscope, coupled with the Raman spectrometer, and observed in different, characteristic locations, as detailed below. Spectral acquisition was replicated at different points, to ensure statistically representative measurements. Raman spectra were recorded using a LabRam microprobe confocal system (Horiba Jobin Yvon GmbH, Kyoto, Japan), equipped with a 632.8 nm, 12 mW He-Ne laser, delivering 7 mW to the analysis spot. A 20× objective was employed, forming a spot of ca. 10 μm at the sample surface. The slit and pinhole of the confocal microscope were set at 200 and 400 μm, respectively, corresponding to a scattering volume of ca. 3 pL. Raman spectra were acquired with a 600 grid mm^−1^ spectrometer. The recorded Raman intensity is proportional to the discharge current of the CCD detector. After optimization of the optical conditions (see next paragraph), spectral acquisition was carried out by averaging 20 spectra, each obtained by accumulating the CCD signal for 60 s, unless otherwise specified. Calibration of the analysis conditions and assessment of possible beam damage under laser probing were carried out for the samples investigated. We tested a range of optical densities (D3, D2, D06, and an unfiltered beam) and assessed changes in fluorescence and spectral pattern, brought about by exposure of the sample to the beam, as detailed below. This methodological assessment is crucial for work with practical materials, containing a high proportion of carbon, that can yield laser-induced fluorescence, liable to impairing spectroscopic capabilities, particularly with the wavelength in the red range, to which the materials of interest are highly responsive [29]. As is detailed in Section 2.3, D06 is enough to quench laser-induced fluorescence, but the limited attenuation might still lead to beam damage. In view of this potential issue, we used graphite samples, since this material is the most sensitive cathode component. Specifically, we employed commercial graphite anodes (FAAM^TM^, 94 wt% graphite SGL synthetic-carbon graphite, 2 wt% carbon black, 2 wt% CMC, and 2 wt% SBR). As is shown in Figure 1—reporting the characteristic carbon G and D bands—repeated spectral measurements each with an exposure of 6 min to the D06-filtered beam gave rise to changes in the spectral pattern, as well as to some residual fluorescence, that were instead not observable with the D2 filter. We thus resorted to filtering with the D2 optical density, in order to completely quench fluorescence, and also in view of the possible formation of additional fluorescent centers as a result of humidity exposure, and to minimize beam damage, though at the cost of longer acquisition times.

### 2.2. Harvesting Cathodic Material from Commercial Batteries

Cathodic materials were extracted from pristine commercial batteries that had just undergone the proprietary formation cycle applied by the manufacturers. LFP cathodes were obtained from A123 ANR26650M1B, 26650 cylindrical format, 2300 mAh, 50 A, 3.3 V, and NMC-LMO cathodes were obtained from SONY US18650V3, 18650 cylindrical format, 2250 mAh, 10 A, 3.7 V. The choice of using widely employed commercial automotive batteries, though at the cost of a lack of complete access to chemical and architectural details, is methodological in nature. In fact, the purpose of this work was to define a full operating pipeline for the use of Raman spectroscopy for the study of practical electrodes, avoiding any form of model materials or working conditions. Battery opening and electrode extraction were carried out following a protocol detailed in the literature, aimed at minimizing material damage [30,31]. Briefly, after discharge to the minimum safe cut-off voltage recommended by the manufacturers, the cells were opened in an MBraun^TM^ argon-filled glove-box by cutting the metal casing with a Dremel^TM^ tool. All subsequent treatments, apart from humidity exposure, were carried out in the same glove-box. After opening, the electrode sheets were unrolled and the cathodes were washed with dimethyl carbonate (DMC, Sigma-Aldrich, St. Louis, MO, USA) to remove residual lithium salts and then left to dry at room temperature. For the present purposes, the classical vacuum-drying process at ca. 80 °C was not necessary, because the we did not mean to carry out in-depth electrochemical characterizations. After drying, the cathode sheets were either cut or punched for subsequent tests. This opening procedure yields materials free from air and humidity damage. The purpose of this procedure is to produce reference materials—that we shall refer to as R-LFP and R-NCM-LMO—to be compared with their counterparts that have undergone humidity damaging. To this aim, the same protocol was applied, but using laboratory ambient conditions, under a fume-hood, instead of operating in a glove-box. The batteries were thus opened in air at ca. 25 °C, with a humidity level of ca. 50%, then vacuum dried at 80 °C overnight and stored in a glove-box. The humidity-exposed cathodes are referred to as H-LFP and H-NCM-LMO. Cathodes for Raman and SEM analyses were removed from the glove-box at the moment of the analysis or were assembled into coin cells for electrochemical characterization inside the glove-box. The composition of R-NCM-LMO was quantified with ICP-OES (ThermoScientific iCAP PRO, Thermo Fisher Scientific Inc., Waltham, MA, USA, courtesy of OMCD Tek Hub S.p.A., Anzola d’Ossola, VB, Italy). Quantitative analysis yielded the following composition: 83.6 ± 0.2% NMC-532, blended with LMO (16.4 ± 0.2%), yielding a theoretical capacity of 144 mAh/g [32].

### 2.3. Ancillary Characterizations

**Figure 2:**
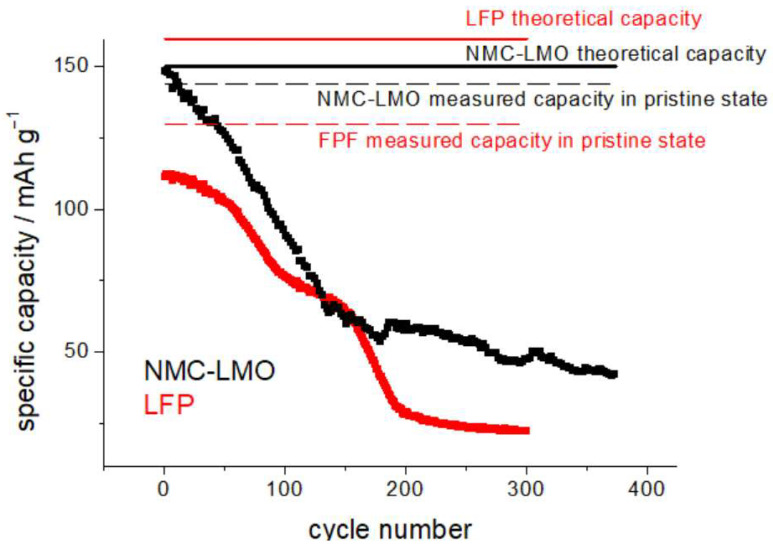
Capacity-fade measurements for H-LFP (red plot) and H-NCM-LMO cathodes (black plot), exposed to humidity (see text for details). CR2032 coin cells cycled at C/10 in the potential intervals 2.5–3.65 V and 3.2–4.25 for LFP (red plots and text) and NCM-LMO (black plots and text), respectively.

SEM images were acquired using a field-emission scanning electron microscope (FE-SEM: Zeiss SUPRA 40, Jena, Germany) operating in a high vacuum. The microscope is equipped with an energy-dispersive X-ray spectrometer (EDS), from the same company. Cathodes without and with humidity exposure were also subjected to cycling in coin cells, to assess the residual capacity and the capacity fade rate. The cathodic material was removed mechanically from one of the sides of the commercial cathode using cotton swabs soaked in N-Methyl-2-pyrrolidone (NMP). Electrochemical tests were carried out with CR2023 coin cells. The cathodes were punched into Ø 10 mm discs, the separator was a glass-fiber foil (WHATMAN GF/A), 250 µm in thickness, soaked with 70 μL 1.0 M LiPF_6_ in EC/DMC = 50/50 (*v*/*v*) (Sigma-Aldrich, St. Louis, MO, USA) electrolyte. As anodes, we employed, without any impact on the relevant results, either Li metal chips (Ø 12 mm, 500 µm thickness) (China Energy Lithium) or Ø 13 mm graphite disks punched from the FAAM^TM^ anode foils mentioned in Section 2.1. Standard stainless-steel casings from Heliume Tech^TM^ (Irvine, CA, USA) were used. All coin cells employed for capacity testing were subjected to a formation cycle consisting of 3 constant current (CC) discharge/charge cycles at C/10 between 2.5 and 3.65 V, and 3.2 and 4.25, for LFP and NCM-LMO, respectively. The C-rate was determined with respect to the theoretical capacities of 160 and 150 mAh g^−1^ for LFP and NCM-LMO, respectively (Figure 2). Both cathode materials exhibit remarkable effects of exposure to humidity, right from the first cycles. LFP exhibits a slower capacity fade over ca. the first 50 cycles, while NMP-LMO exhibits an immediate and drastic drop. This behavior is coherent with that reported in the literature [12,16,33,34,35].

**Figure 3:**
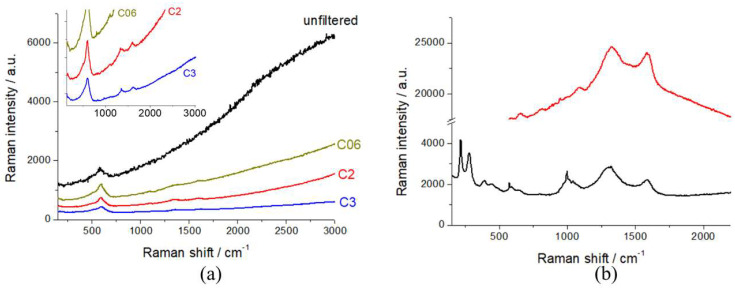
Raman spectra of (**a**) R-NMC-LMO (inset: magnification of the low-intensity region of the main panel) and (**b**) R-LFP cathodes, measured with the laser beam filtered with the indicated optical densities.

In order to assess the best working conditions for Raman spectroscopy, as put forward in Section 2.1, we calibrated the laser intensity with a series of optical densities. In Figure 3, we report the Raman spectra of R-NMC-LMO and R-LFP, measured with the unfiltered beam and with selected optical densities. It can be noticed that the unfiltered beam yields a high level of laser-induced fluorescence from the conductivity additive, that covers the vibrational features of the cathodic active material, that become visible with and optical density of D06. In order to exclude beam damage, as detailed in Section 2.1, all spectroscopy work was carried out with a D2 filter.

## 3. Results and Discussion

### 3.1. Structural and Morphological Modifications of Humidity-Exposed LFP Cathodes

**Figure 4:**
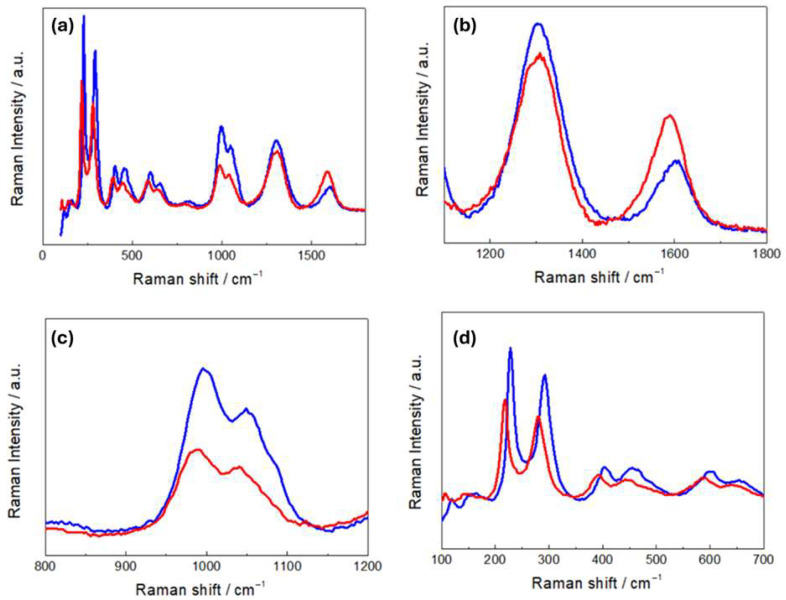
(**a**) Raman spectra of pristine (R-LFP, blue curves) and humidity-damaged (H-LFP, red curves) LFP electrodes. Details of (**b**) the carbon bands; (**c**) the PO_4_^3−^ stretching bands; (**d**) the O-P-O bending modes and vibrations related to Li- and Fe-related bonds.

Figure 4a compares the Raman spectra of R- and H-LFP electrodes. These vibrational spectra are characterized by two main groups of bands: (i) a sequence of peaks in the range 100–1100 cm^−1^,which is characteristic of LFP, and (ii) the couple of peaks at ca. 1350 and 1600 cm^−1^, corresponding to the D and G carbon bands, respectively. The LFP bands include modes associated with PO_4_^3−^ and the coupled motion of Fe^2+^ and PO_4_^3−^ [36]. The Raman modes in the range 990–1080 cm^−1^ are attributed to the PO_4_^3−^ unit and involve symmetric and asymmetric stretching of P–O bonds [37]. Modes at 626 and 587 cm^−1^ correspond to the symmetric (ν_2_) and anti-symmetric (ν_4_) bending of the O–P–O angles, respectively. The mode at 395 cm^−1^ is associated with the lithium cage and oxygen ion breathing modes, while modes in the 100–300 cm^−1^ range arise from Fe translation and coupled translation/vibration of Fe and PO_4_^3−^ [38,39]. The G band at 1600 cm^−1^ corresponds to the E_2_g active mode of graphite, while the D band at 1350 cm^−1^ is assigned to the A_1_g mode and is associated with symmetry breaking at the edges of graphite sheets [31]. As far as the graphite-related peaks are concerned, (Figure 4b), the spectrum of the H-LFP electrode shows a blue shift in the D and G bands with respect to that of the R-FFP electrode. The blue shift of these bands is a well-documented phenomenon in LIB cycling, that is associated with structural and chemical changes in the carbon matrix. In fact, chemical and electrochemical stress can introduce defects and disorders into the carbon matrix, such as vacancies and edge defects, and can generate new functional groups. These defects tend to disrupt the sp^2^ hybridized carbon network, leading to changes in the Raman-active vibrational modes. An increase in disorder is typically accompanied by a blue shift in the D band and changes in the D/G intensity ratio [40]. Moreover, strain can lead to a blue shift in the G band due to stiffening of the C–C bonds, corresponding an alteration of the electronic structure of the carbon [41]. Finally, SEI formation has been reported to lead to changes in the local bonding environment of the carbon surface, that yields blue shifts [42]. In the Raman shift range corresponding to the PO_4_^3−^ stretching modes (Figure 4c), the R-LFP electrodes exhibit the typical spectral pattern of LFP. Conversely, the H-LFP sample displays, in addition to the band at 993 cm^−1^, indicative of LFP lithiation, a shoulder at 957 cm^−1^, corresponding to the symmetric PO_4_^3−^ stretching vibration, characteristic of delithiated LFP [43]. Finally, in the O-P-O- bending region (Figure 4d), a weak band at 441 cm^−1^ appears in the spectral pattern of the water-exposed electrode, which is absent in the spectra of pristine LFP, that is attributable to imperfect lithiation [44].

**Figure 5:**
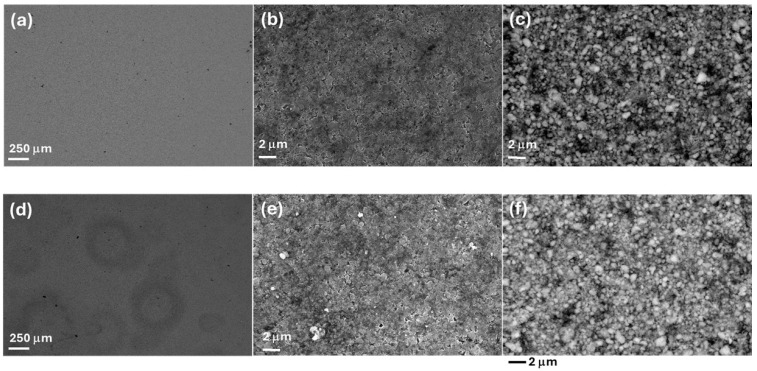
SEM micrographs (**a**–**d**) of LFP electrodes in pristine (R-LFP) and humidity-damaged (H-LFP. (**a**–**c**) SEM of R-LFP, (**d**–**f**) SEM of H-LFP. (**a**,**b**,**d**,**e**) Secondary electron images, (**c**,**f**) backscattered electron images.

SEM micrographs of pristine (R-LFP) and humidity-damaged (H-LPF) LFP cathodes are reported in panels (a–f) of Figure 5. The low-magnification micrographs of panels (a) and (d) highlight the formation of damaging patterns at the H-LFP mesoscopic scale. Moreover, images at a higher magnification (panels (b), (c), (e) and (f)) highlight that exposure to humidity leads to a less defined micro texture of the LFP clusters. Specifically, H-LPF (panels (e) and (f)) exhibits an etched morphology characterized by looser and spheroidized particles, suggesting some degree of corrosive attack and the formation of a surface layer, in-keeping with the literature [16,45], that reports relative LFP tolerance to water traces in the electrolyte. These features are the morphological counterpart of the structural alterations highlighted by Raman spectroscopy [46,47,48].

### 3.2. Structural and Morphological Modifications of Humidity-Exposed NMC-LMO Cathodes

**Figure 6:**
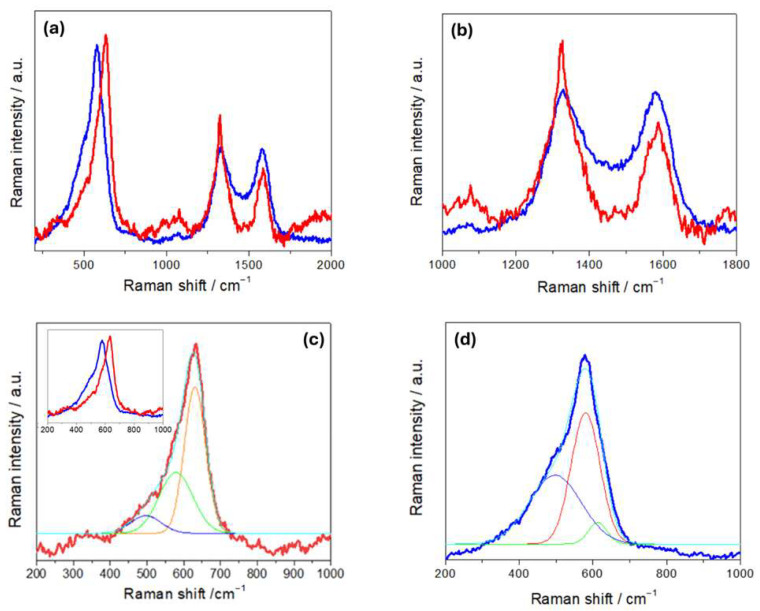
Raman spectra of NMC-LMO electrodes in pristine (R-NMC-LMO, blue curves) and humidity-damaged (H-NMC-LMO, red curves) conditions. Details of (**a**,**b**) the carbon bands and (**c**,**d**) the NCM-LMO stretching bands with respective fits: the two spectra are overlapped in the inset of panel (**c**) (see text for details).

The Raman spectra of R- and H-NMC-LMO electrodes (Figure 6) are characterized by a set of bands corresponding to NMC and LMO modes in the range 450–650 cm^−1^, and the D and G carbon modes at 1350 and 1600 cm^−1^. The LMO spectrum exhibits a strong band at 625 cm^−1^, a shoulder at approximately 570 cm^−1^ and two weak bands at approximately 470 cm^−1^ and 360 cm^−1^ [49]. The band at 625 cm^−1^ can be attributed to symmetric stretching vibrations of the MnO_6_ octahedra, while the band at 570 cm^−1^ is associated with vibrations of Mn^IV^-O bonds. Raman spectra of NMC are characterized by strong bands located at 494, 597, and 630 cm^−1^. The features at 494 and 597 cm^−1^ can be attributed to the Co-O vibration, while the feature at 630 cm^−1^ corresponds to Mn-O vibrations [26]. Other studies also reported bands at 470 and 530 cm^−1^, assigned to Ni-O vibrations [50].

In Figure 6b, we found that, on the one hand, the D and G bands of H-NMC-LMO exhibit a blue and a red shift, respectively, and on the other hand, the band intensity ratios between the D band and the G band changed from I_D_/I_G_ 1.71 for R-NMC-LOMO to 1.02 for H-NMC-LOMO. As far the blue shift is concerned, these differences can be explained along the same lines of Section 3.1. Instead, on the basis of the literature, the red shift can be explained with the same kind of damaging mechanisms, activating different specific pathways [51,52,53]. An exact mechanistic assignment is beyond the scope of the present study, but the diagnostic value of relative spectral variations is clear. Similar comments apply to band ratio modifications: in particular, variations in the D/G intensity ratio denote differences in structural disorder or defect concentration within the carbon matrix, that in the literature have been referred to as different types of damaging modes [40,54,55,56]. In addition, a broad peak at ca. 1077 cm^−1^ is observed only in R-NMC-LMO. This vibrational feature is characteristic of carbonates, like NiCO_3_, Li_2_CO_3_, CoCO_3_, and MnCO_3_, that can be related to CEI formation [4]. The absence of these vibration in the H-NMC-LMO can be explained with their leaching by HF produced in the reaction of the electrolyte with humidity. Panels (c) and (d) of Figure 6 report the fitting of the M-O vibrational band. The band of R-NMC-LMO can be fitted with three peaks centered at 497, 577, and 631cm^−1^, while that of H-NMC-LMO has three components at 496, 579, and 612 cm^−1^. For the R-NMC-LMO cathode, the peaks at 631 and 577 cm^−1^ can be assigned to Mn-O vibrations of either NMC or LMO, while the vibration at 496 cm^−1^ corresponds to a Co-O vibration of NMC [4]. The same peaks at 577 and 496 cm^−1^ are also found in the H-NMC-LMO sample that, in addition, shows differences in the spectral pattern. Specifically, the peak at 630 cm^−1^, corresponding to a Mn-O mode, disappears; this is probably due to Mn dissolution from LMO, which is very sensitive to HF [57]. Instead, a new peak appears at 612 cm^−1^, which can be attributed to hydrated nickel salts and carbonates [4]. The results of Raman measurements are thus diagnostic of the expected acid-induced dissolution scenario caused by exposure to humidity.

**Figure 7:**
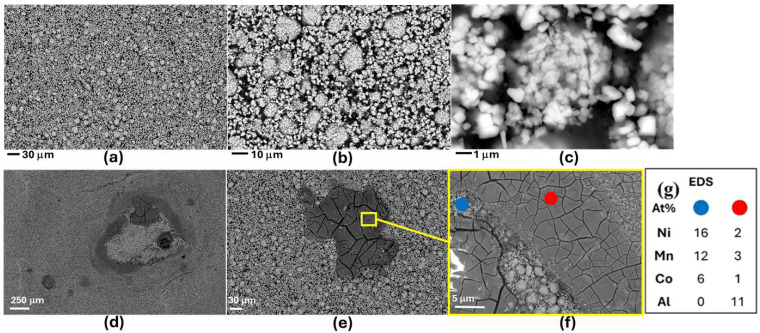
Secondary-electron SEM micrographs of NMC-LMO electrodes in pristine (R-NMC-LMO, panels (**a**–**c**)) and humidity-damaged (H-NMC-LMO, panels (**d**–**e**)) conditions. (**g**) EDS compositional analysis H-NMC-LMO.

The SEM micrographs of Figure 7 show a notable impact of humidity exposure on the morphology of NMC-LMO cathodes, in-keeping with the information reported in the literature [16,58,59,60]. In particular, totally different features appear for R- and H-NMC-LMO samples, characterized by different length scales. Such a drastic morphology transformation is characteristic of the formation of a compact film of corrosion products, which covers the original granular structure. EDS mapping (Figure 7g), considering the penetration depth of the probe, hints at local Mn loss and accumulation of Al deriving from current-collector corrosion. Elemental mapping of the damaged surface (red spot), compared to the pristine surface (blue spot), reveals a higher concentration of F and Al, along with a reduced concentration of Mn. The detected F is likely associated with reaction products brought about by HF formation during battery opening, while Al derives from current-collector corrosion. The morpho-chemical differences between R- and H-NCM/LMO electrodes are the counterpart of the structural alterations highlighted by Raman spectroscopy, as seen in Figure 6.

## 4. Conclusions

This study offers novel experimental and methodological information on the use of Raman spectroscopy for the investigation of practical battery materials, applied to the case of the structural impact of humidity exposure of cathode composites based on lithium iron phosphate (LFP) and nickel–manganese–cobalt/lithium–manganese oxide (NMC-LMO). Apart from the specific case of NMC-LMO, which does not seem to have been investigated specifically before this contribution of ours, the materials-science topic has been extensively covered, but a detailed description of the concrete experimental factors enabling Raman studies of this class of materials is still missing. After having provided rational guidelines for the achievement of sound spectral quality, we have shown that vibrational spectroscopy can pinpoint specific structural effects of humidity-induced degradation that clearly correlate with morphological and compositional alterations, in turn corresponding to capacity loss. Specifically, in NMC-LMO, where compact corrosion films, Mn dissolution, and CEI alterations were observed, Raman spectroscopy confirmed the disappearance of characteristic metal–oxygen vibrational modes and the appearance of new peaks related to acid-driven reactions and the formation of hydrated byproducts. In contrast, LFP, displaying more resilience to humidity exposure, retains much of its structural integrity and electrochemical performance. These findings, on the one hand, further stress the material-specific nature of humidity-induced damage and, on the other hand, emphasize the diagnostic value of multi-technique approaches for the rational understanding of battery-material degradation pathways.

## Data Availability

The original contributions presented in this study are included in the article. Further inquiries can be directed to the corresponding author.
